# A new species of *Galleria* Fabricius (Lepidoptera, Pyralidae) from Korea based on molecular and morphological characters

**DOI:** 10.3897/zookeys.970.54960

**Published:** 2020-09-21

**Authors:** Seung Jin Roh, Haechul Park, Seong-Hyun Kim, So-Yun Kim, Yong-Su Choi, Jeong-Hun Song

**Affiliations:** 1 Department of Agricultural Biology, National Institute of Agricultural Sciences, Wanju 55365, South Korea Department of Agricultural Biology, National Institute of Agricultural Sciences Wanju-gun South Korea

**Keywords:** cryptic species, Galleriinae, new species, plastic eating moth, Pyraloidea, wax worms

## Abstract

The greater wax moth, *Galleria mellonella* Linnaeus, is well known as a pest of honey bees and for the biodegradation of wax and polyethylene by their larvae. The genus *Galleria* has long been considered monotypic and found worldwide. A taxonomic study of the genus *Galleria* is presented based on morphological and molecular characters (*COI*, *CAD*, *wg*). A new species (*Galleria similis* Roh & Song, **sp. nov.**) is recognized on the Korean peninsula. The new species is superficially similar to *G. mellonella* but they can be separated by the structures of hindwing venation and male genitalia. Habitus photographs and illustrations of diagnostic characters are provided.

## Introduction

The family Pyralidae is large group of Lepidoptera, placed in the superfamily Pyraloidea consisting of 1055 genera with 5921 described species (van Nieukerken et al. 2011). A molecular phylogeny and revised classification of the Pyralidae recognized five subfamilies, Chrysauginae, Epipaschiinae, Galleriinae, Phycitinae, and Pyralinae (Regier et al. 2012).

Among the Galleriinae, the monotypic genus *Galleria* Fabricius, 1798 was established with the type species *Phalaena cereana* Blom, 1764. *Galleria mellonella* (Linnaeus) is a ubiquitous pest of honey bees, *Apis mellifera* Linnaeus and *A. cerana* Fabricius (Ellis et al. 2013; Kwadha et al. 2017). They live on honeycomb in beehives, feeding on honey, beeswax, and the skin of bee pupae (Oldroyd 1999, 2007; Martel et al. 2006; Klein et al. 2007; Kong et al. 2019). Recent studies have shown that the larvae have the ability to biodegrade polyethylene in their guts (Yang et al. 2014; Bombelli et al. 2017; Kong et al. 2019).

The genus *Galleria* is superficially similar to the genus *Achroia* Hübner, 1819 (Kwadha et al. 2017), but can be distinguished from the latter by the presence of four stemmata on the head of the larva, concaved in the termen of the forewing, and the Cu vein apparently four-branched from the hindwing (Ellis et al. 2013).

In this paper, we describe *Galleria similis* Roh & Song, sp. nov. based on morphological and molecular characters, and provide habitus photographs and illustrations of diagnostic characters for identification of the two species of the genus *Galleria*.

## Materials and methods

The material examined in this study is deposited in the Systematic Entomology Laboratory, National Institute of Agricultural Sciences (NAS), Wanju, Korea. Specimens were dissected and examined after mounting on glass slides; male genitalia in 60% Euparal and wing venation based on dried specimens. Photographs of adults and male genitalia were taken using a Dhyana 95 scientific CMOS camera (Tucsen, Fuzhou, China) attached to a Leica DM 2000 LED optical microscope (Leica, Wetzlar, Germany). Terminology for morphological characters of the adult follow Smith (1965).

Genomic DNA from four specimens of *Galleria similis* and 19 specimens of *G. mellonella* was extracted from the legs of dried specimens of adults in 100% alcohol using a MagListo 5M Genomic DNA Extraction Kit (Bioneer Corporation, Daejeon, Republic of Korea) according to the manufacturer’s protocol. One mitochondrial protein coding gene, the cytochrome oxidase subunit I gene (*COI*) (Folmer et al. 1994) and two nuclear protein coding genes, Carbamoyl-phosphate synthetase 2, Aspartate transcarbamylase, and Dihydroorotase (*CAD*) and Wingless (*wg*) were sequenced (Haines and Rubinoff 2012) (Table 1). Primers and amplification strategies followed Haines and Rubinoff (2012) and are detailed in Table 2. PCR conditions for amplification followed Haines and Rubinoff (2012), and directly sequenced at Macrogen (Geumcheon-gu, Seoul, Korea). Contigs were assembled in Geneious prime (Kearse et al. 2012). Successful *COI*, *CAD* and *Wingless* sequences were uploaded to GenBank (Table 1).

**Table 1.** *Galleria* species and their *COI* barcodes and nuclear protein coding gene sequences with their associated and GenBank accession numbers as used in this study. Dashes indicate missing data.

Species	Voucher No.	*COI*	*CAD*	*wg*
*Galleria mellonella*	15310	MT439336	MT447104	MT447124
	15311	MT439337	MT447105	MT447125
	15312	MT439338	MT447109	MT447126
	15313	MT439349	MT447106	MT447127
	15314	MT439350	MT447107	MT447128
	15616	MT439351	MT447110	MT447129
	15617	–	MT447108	MT447130
	21361	MT439339	–	MT447131
	21362	MT439340	MT447111	MT447132
	21363	MT439341	MT447115	MT447133
	21364	MT439342	MT447114	MT447134
	21365	–	MT447119	MT447135
	21412	MT439343	MT447116	MT447136
	21413	MT439352	–	–
	21414	MT439346	MT447112	MT447137
	21415	MT439344	MT447113	MT447138
	21416	MT439347	MT447120	MT447139
	21417	MT439345	MT447118	MT447140
	21418	MT439348	MT447117	MT447141
*G. similis*	15315	MT447100	MT447121	MT447142
	21366	MT447101	MT447122	MT447143
	21367	MT447102	MT447123	MT447144
	21368	MT447103	–	MT447145

**Table 2.** List of primers and amplification strategies used in this study (abbreviations: s = second, min = minute).

Genes	Primers	Sequences (5' to 3')	Amplification strategies
*COI*	LCO1490	GGTCAACAAATCATAAAGATATTGG	LCO1490 + HCO2198 (Folmer et al. 1994)
	HCO2198	TAAACTTCAGGGTGACCAAAAAATCA	
*CAD*	CAD4_Pyr_F	GAAGAAGCATTTCAAAAAGC	CAD4_Pyr_F + CAD4_Pyr_R (Haines and Rubinoff 2012)
	CAD4_Pyr_R	CKRTCACTCATGTCRTA	
*wg*	LepWg1	GARTGYAARTGYCAYGGYATGTCTGG	LepWg1 + LepWg2 (Brower and Desalle 1998)
	LepWg2	ACTICGCARCACCARTGGAATGTRCA	

* PCR amplifications condition - *COI*: 5-min 95 °C; 35 cycles: 30-s 95 °C, 25-s 48 °C, 45-s at 72 °C; 5-min 72 °C. - *CAD*: 2-min 94 °C, 1-min 50 °C, 1-min 72 °C; 34 cycles: 1-min 94 °C, 1-min 50 °C, 1-min at 72 °C; 12-min 72 °C. - *wg*: 2-min 94 °C, 1-min 56 °C, 1-min 72 °C; 34 cycles: 1-min 94 °C, 1-min 56 °C, 1-min at 72 °C; 12-min 72 °C.

The barcodes were compared to 93 DNA barcodes of the genera *Galleria* and *Achroia* downloaded from BOLD systems v4 (BIN numbers: BOLD:AAA0965, BOLD:AAL2955, BOLD:ACO9701). A neighbor-joining analysis (NJ) was performed with MEGA X (Kumar et al. 2018) using the Kimura-2-Parameter (K2P) model (Kimura 1980) for nucleotide substitutions. Bootstrap support values for each node were also evaluated via MEGA X with 1000 replicates. Parsimony analyses (PA) with bootstrap were conducted in TNT 1.5 (Goloboff and Catalano 2016) using search strategies described by Song and Ahn (2018).

Intra- and inter-specific distances in different taxonomic levels were calculated using the uncorrected pairwise distance method (Srivathsan and Meier 2012). To explore molecular diagnostic characters for the *Galleria* species, we used the “list common synapomorphies” function of TNT and then examined thoroughly listed characters in the alignment file.

## Results

### Molecular character analysis

A total of 21 new sequences was generated from four specimens of *Galleria similis* and 17 specimens of *G. mellonella* (524–650 bp of partial *COI* barcode region, 613 bp of partial *CAD*¸ and 432 bp of partial *wg* gene region). All new sequences were uploaded to GenBank (Table 1). The DNA barcodes (*COI*) were compared to those of 72 DNA barcodes in 16 countries (*G. mellonella*), one Australian specimen (*Galleria* sp.) and seven lesser wax moths (*Achroia grisella* Fabricius) downloaded from BOLD systems v4 (Fig. 7).

Genetic divergence of *COI* using uncorrected *p*-distance among the *Galleria* and *Achroia* species ranged from 5.3% to 12.0%, while intraspecific divergence ranged from 0% to 2.2% (Table 3). All four species were strongly supported as a single lineage on both NJ and PA trees (Figs 7, 8). The molecular analyses (*p*-distance, NJ and PA analyses) revealed that *G. mellonella* was closely related to *G. similis* (Table 3; Figs 7, 8). The maximum difference among populations within *G. mellonella* was 2.2%, and within *G. similis* was 0% (Table 3). For these two species, it is difficult to correctly delimit each species, due to their extreme similarities in external morphological characters (see taxonomy section below). In contrast to morphological characters, however, genetic divergence strongly supported the separation of *G. mellonella* and *G. similis*. The minimum inter-specific difference between the two species (5.3%) was much higher than the maximum intraspecific difference of *G. mellonella* (2.2%) (Table 3). Furthermore, molecular diagnostic characters for the *Galleria* species, *G. mellonella* and *G. similis* contained 15 characters for *COI*, one character of *CAD* and four characters of *wg* gene regions (Table 4).

**Table 3.** Inter- and intraspecific genetic differences in the two genera *Galleria* and *Achroia* species for *COI* (658 bp) calculated using *p*-distance.

	***G. mellonella***	***G. similis***	***Galleria* sp.**	***A. grisella***
*G. mellonella*	0–0.022			
*G. similis*	0.053–0.066	0		
*Galleria* sp.	0.112–0.119	0.114	0	
*A. grisella*	0.107–0.116	0.117–0.119	0.116–0.120	0–0.003

**Table 4.** List of 20 molecular diagnostic characters used to determine the genetic distinctiveness of two cryptic species *Galleria mellonella* and *G. similis* based on mtDNA partial *COI*, nuDNA partial *CAD* and *wg* gene region. Numbers indicate nucleotide sites in the sequenced 658 bp portion of the *COI* gene, 613 bp portion of the *CAD* gene and 432 bp portion of the *wg* gene. Number position follows *G. mellonella*: MT439366 (*COI*), MT447104 (*CAD*) and MT447124 (*wg*).

**Species**	**Genes**									
	***COI***									
	16	34	109	197	232	259	271	274	280	307
*G. mellonella*	T	A	A	T	T	T	T	T	T	T
*G. similis*	C	T	G	C	C	C	C	C	C	C
**Species**	***COI***					***CAD***	***wg***			
	385	391	403	424	470	319	129	241	343	379
*G. mellonella*	T	T	C	T	T	G	A	T	C	C
*G. similis*	C	C	T	C	C	A	C	C	T	T

We also found three distinct differences in the amino acid sequences of each protein (Table 5). In particular, the transition from G (guanine) to A (adenine) at the 319 site of *CAD* protein led to a change from a hydrophobic amino acid (Alanine, A) to a hydrophilic amino acid (Threonine, T), and the transversion from A to C (cytosine) at the 129 site of the wg protein led to a change from a hydrophilic amino acid (Glutamate, E) to a hydrophobic amino acid (A). The molecular characters provided further evidence that new species *G. similis* was distinct and valid.

**Table 5.** List of three molecular diagnostic characters used to determine the molecular distinctiveness of two cryptic species *Galleria mellonella* and *G. similis* based on amino acid sequences of partial *COI*, *CAD*, and *wg* protein region. Numbers indicate amino acid site in the sequenced 201 amino acid (aa) portion of the *COI* protein, 204 aa portion of the *CAD* protein and 143 aa portion of the *wg* protein. Number position follows *G. mellonella*: the translated amino acid sequences of MT439366 (*COI*), MT447104 (*CAD*) and MT447124 (*wg*).

**Species**	**Proteins**		
	***COI***	***CAD***	***wg***
	152	107	43
*G. mellonella*	V	A	E
*G. similis*	I	T	A

### Taxonomic accounts

#### Genus *Galleria* Fabricius, 1798

*Galleria* Fabricius, 1798: 419, 462. Type species: *Phalaena cereana* Blom, 1764, by subsequent designation by Latreille (1810: 441).

*Cerioclepta* Sodoffsky, 1837: 93. Type species: *Galleria mellonella* Linnaeus, 1758, by original designation.

*Vindana* Walker, 1866: 1706. Type species: *Vindana obliquella* Walker, 1866, by monotypy.

##### Galleria similis

Taxon classificationAnimaliaLepidopteraPyralidae

Roh & Song sp. nov.

CEC8350B-700F-53BD-A9FE-574CA0A3D0C6

http://zoobank.org/DD9DF8D5-D3D5-4235-80AE-294C9B731EAB

Figures 2 , 4 , 6

###### Type material.

***Holotype.*** ♂, **Korea**: Wanju-gun, 14.xi.2014, 35°49'45.64"N, 127°02'27.20"E, leg. H.S. Shim, genitalia slide no. 15315, DNA barcode GenBank accession no. MT447100 (NAS). ***Paratypes.*** 3♂, **Korea**: Tongyeoung, 17.i.2020, 34°50'58.58"N, 127°26'51.79"E, leg. J.-H. Song, genitalia slide no. 21366–21968, DNA barcode GenBank accession no. MT447101, MT447102, and MT447103 (NAS).

###### Diagnosis.

**Figures 1, 2.**
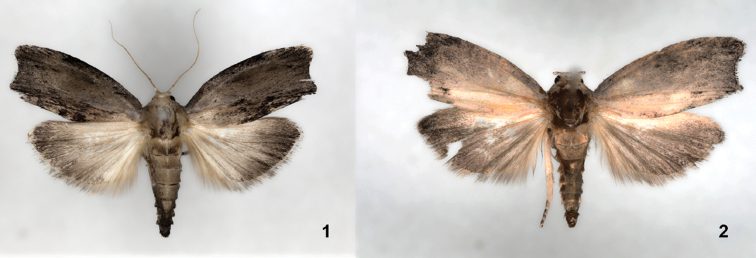
Adults of *Galleria* species. **1** Male of *G. mellonella***2** male of *G. similis*, holotype.

**Figures 3, 4.**
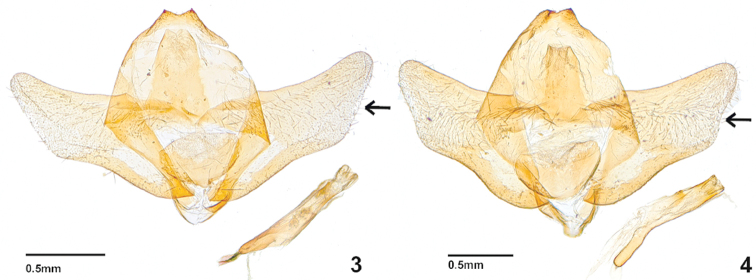
Male genitalia of *Galleria* species. **3***G. mellonella* (slide no. 21364) **4***G. similis*, paratype (slide no. 21367).

**Figures 5, 6.**
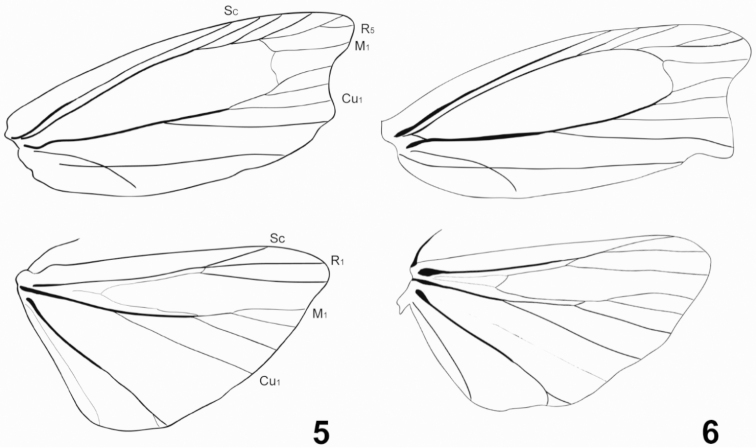
Male wing venation of *Galleria* species. **5***G. mellonella***6***G. similis*, paratype.

*Galleria similis* sp. nov. (Figs 2, 4, 6) is very similar to *G. mellonella* (Figs 1, 3, 5) but can be distinguished by a square discal cell of its hindwing venation (Fig. 6) and the different shape of male genitalia (Fig. 4, *G. similis*: valva shorter and wider, concave at outer margin). *Galleria similis* sp. nov. had 15, one and four diagnostic characters from 658 bp of partial *COI*, 613 bp of partial *CAD* and 423 bp of partial *wg* gene region, respectively (Table 4). Our study showed that morphological and molecular characters can be used to resolve the status of cryptic species, *G. mellonella* and *G. similis*. A cryptic species was suggested by the unusually high genetic distances within specimens originally identified as *G. mellonella*.

###### Description.

***Adult.* Male** (Fig. 2). Head: vertex densely clothed with gray hair-like scales; labial palpus three-segmented. Thorax: Light brown; notum covered with gray scales. Legs with femora, tibiae, and tarsi clothed with light gray piliform scales; tarsi apical and medial spurs covered dark-brown scales. Wingspan 21.5–32.0 mm. Forewing (Fig. 6) narrow, costa straight at base and gently curved beyond 4/5, termen concave; tornus pointed, 9 separate veins originating at the discal cell; Sc terminating at 4/5 costa; R_5_ originated at R_4_, M_1_ and M_2_ parallel; M_2_, M_3_ originating at distal corner of discal cell; Cu_1_ and Cu+A_1_ parallel, ground color yellowish white with gray and some dark overscaling. Hindwing (Fig. 6) discal cell square, L/W ratio 1.72; costa straight, apex straightly curved to termen; Sc straight to 3/5 costa; R_1_, R_2_ and R_3_ present; R_1_ and R_2_ terminating at apex; M_2_ originating at 1/5 M_3_; CuA_1_ and CuA_2_ parallel; A_1_ originating at 4/5 Cu_2_. Hindwing covered with dark-brown scales; postmarginal part present with short light brown hairs. Abdomen: Male genitalia (Fig. 4) with uncus concave and hooked; tegumen wide at base; gnathos long; valva short and wide, costa straight, termen relatively concave, small setae present sparsely on outer and inner surface; vinculum narrower than gnathos; juxta heart shaped; saccus very short and slender; phallus slightly short and thick, vesica with short setae, ductus ejaculatorius present.

**Female.** Unknown.

###### Distribution.

Korea.

###### Etymology.

Named from the Latin *similis* meaning “similar”, which refers to the similar morphological characters with *G. mellonella*.

**Figure 7.**
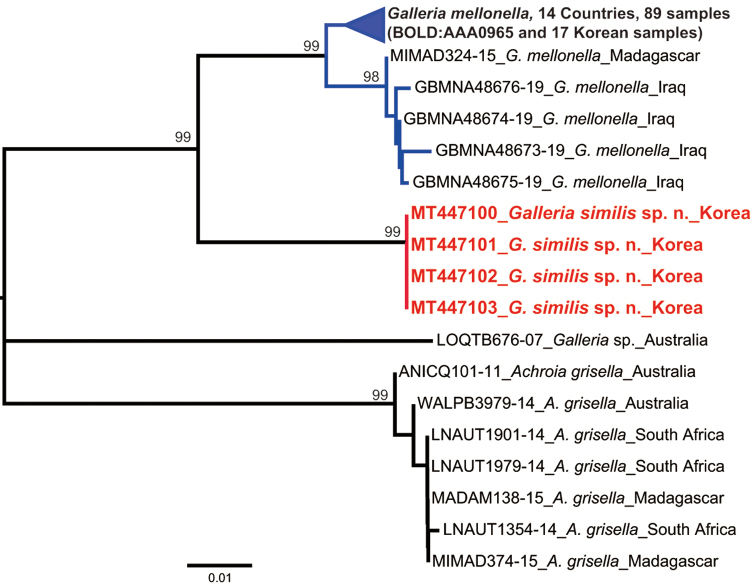
Neighbor-Joining tree based on partial *COI* gene sequences with bootstrap values. Scale bar indicates the expected number of substitutions per site.

**Figure 8.**
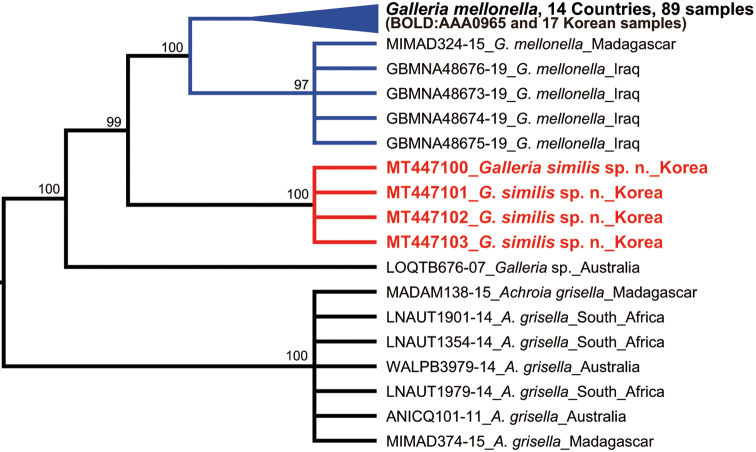
Strict consensus tree of equally parsimonious cladograms based on partial *COI* gene sequences with bootstrap values.

## Supplementary Material

XML Treatment for Galleria similis

## References

[B1] Blom CM (1764) Beskrifning pa en liten fjaril, som utoder Bi-Stockar. Kungliga Svenska Vetenskapsakademiens Handlingar 25: 12–18.

[B2] Bombelli P Howe CJ Bertocchini F (2017) Polyethylene bio-degradation by caterpillars of the wax moth *Galleria mellonella* Current Biology 27: R283–R293. 10.1016/j.cub.2017.02.060 28441558

[B3] Brower AVZ Desalle R (1998) Patterns of mitochondrial versus nuclear DNA sequence divergence among nymphalid butterflies: the utility of wingless as a source of characters of phylogenetic inference. Insect Molecular Biology 7: 73–82. 10.1046/j.1365-2583.1998.71052.x 9459431

[B4] Ellis JD Graham JR Mortensen A (2013) Standard methods for wax moth research. Journal of Apicultural Research 52: 1–17. 10.3896/IBRA.1.52.1.10 PMC3816652 24198438

[B5] Fabricius JC (1793) Entomologia systematica emendata et aucta. Secundum classes, ordines, genera, species, adiectis synonimis, locis, observationibus, descriptionibus. Vol. Tome III, Pars I, C.G. Proft, Fil. et Soc., Hafniae, 1–488. 10.5962/bhl.title.125869

[B6] Fabricius JC (1794) Entomologica systematica emendata et aucta. Secundum classes, ordines, genera, species adiectis synonymis, locis, observationibus, descriptionibus. C.G. Proft et C. F. Mohr, Hafniae et Kiliae, 1–349.

[B7] Fabricius JC (1798) Supplementum Entomologiae Systematicae. Proft et Storch, Hafniae, [i]-[iv], 1–572, 1–52.

[B8] Folmer O Black M Hoeh W Lutz R Vrijenhoek R (1994) DNA primers for amplification of mitochondrial cytochrome c oxidase subunit I from diverse metazoan invertebrates. Molecular Marine Biology and Biotechnology 3: 294–299. 7881515

[B9] Goloboff PA Catalano SA (2016) TNT version 1.5, including a full implementation of phylogenetic morphometrics. Cladistics 32: 221–238. 10.1111/cla.12160 34727670

[B10] Haines WP Rubinoff D (2012) Molecular phylogenetics of the moth genus *Omiodes* Guenée (Crambidae: Spilomelinae), and the origins of the Hawaiian lineage. Molecular Phylogenetics and Evolution 65: 305–316. 10.1016/j.ympev.2012.06.021 22772027

[B11] Hübner J (1800–1838) c: Sammlung europäischer Schmetterlinge. Horde 3. Bombyces-Spinner [continued by C. Geyer], Augsburg, 101–154.

[B12] Kearse M Moir R Wilson A Stones-Havas S Cheung M Sturrock S Buxton S Cooper A Markowitz S Duran C Thierer T Ashton B Meintjes P Drummond A (2012) Geneious basic: an integrated and extendable desktop software platform for the organization and analysis of sequence data. Bioinformatics 28: 1647–1649. 10.1093/bioinformatics/bts199 PMC3371832 22543367

[B13] Kimura M (1980) A simple method for estimating evolutionary rates of base substitutions through comparative studies of nucleotide sequences. Journal of Molecular Evolution. 16: 111–120. 10.1007/BF01731581 7463489

[B14] Klein AM Vaissiere BE Cane JH Steffan-Dewenter I Cunningham SA Kremen C Tscharntke T (2007) Importance of pollinators in changing landscapes for world crops. Proceedings Royal Society London B 274: 303–313. 10.1098/rspb.2006.3721 PMC1702377 17164193

[B15] Kong HK Kim HH Chung JH Jun JH Lee S Kim HM Jeon S Park SG Bhak J Ryu CM (2019) The *Galleria mellonella* Hologenome Supports Microbiota-Independent Metabolism of Long-Chain Hydrocarbon Beeswax Cell Reports 26: 2451–2464. 10.1016/j.celrep.2019.02.018 30811993

[B16] Kumar S Stecher G Li M Knyaz C Tamura K (2018) MEGA X: Molecular Evolutionary Genetics Analysis across computing platforms. Molecular Biology and Evolution 35: 1547–1549. 10.1093/molbev/msy096 29722887 PMC5967553

[B17] Kwadha CA Ong’amo GO Ndegwa PN Raina SK Fombong AT (2017) The Biology and Control of the Greater Wax Moth, *Galleria mellonella*. Insects 733: 49–64. 10.3390/insects8020061 PMC5492075 28598383

[B18] Latreille PA (1810) Consid érations g énérales sur l’ordre naturel des animaux composant les classes des crustac és, des arachnides, et des insectes; avec un tableau m éthodique de leurs genres, dispos és en familles. Schoell, Paris, 1–444. 10.5962/bhl.title.13342

[B19] Linnaeus C (1758) Systema naturae per regna tria naturae, secundum classes, ordines, genera, species, cum characteribus, differentiis, synonymis, locis.’ Laurentii Salvii, Holmiae, 1–824. 10.5962/bhl.title.542

[B20] Martel AC Zeggane S Drajnudel P Faucon JP Aubert M (2006) Tetracycline residues in honey after hive treatment. Food Additives & Contaminants 23: 265–273. 10.1080/02652030500469048 16517528

[B21] Nieukerken EJ van Kaila L Kitching IJ Kristensen NP Lees DC Minet J Mitter C Mutanen M Regier JC Simonsen TJ Wahlberg N Yen SH Zahiri R Adamski D Baixeras J Bartsch D Bengtsson BA Brown JW Bucheli SR Davis DR De Prins J De Prins W Epstein ME Gentili-Poole P Gielis C Hattenschwiler P Hausmann A Holloway JD Kallies A Karsholt O Kawahara AY Koster SJC Kozlov MV Lafontaine JD Lamas G Landry JF Lee S Nuss M Park KT Penz C Rota J Schitlmeister A Schmidt BC Sohn JC Solis MA Tarmann GM Warren AD Weller S Yakovlev RV Zolotuhin VV Zwick A (2011) Order Lepidoptera Linnaeus, 1758. In: Zhang ZQ (Ed.) Animal biodiversity: an outline of higher-level classification and survey of taxonomic richness.Zootaxa 3148: 212–221. 10.11646/zootaxa.3148.1.41

[B22] Oldroyd BP (1999) Coevolution while you wait: *Varroa jacobsoni*, a new parasite of western honeybees. Trends in Ecology & Evolution 14: 312–315. 10.1016/S0169-5347(99)01613-4 10407428

[B23] Oldroyd BP (2007) What’s Killing American Honey Bees?. PLOS biology 5: 1195–1199. 10.1371/journal.pbio.0050168 PMC1892840 17564497

[B24] Regier JC Mitter C Solis MA Hayden JE Landry B Nuss M Simonsen TJ Yen SH Zwick A Cummings MP (2012) A molecular phylogeny for the pyraloid moths (Lepidoptera: Pyraloidea) and its implications for higher-level classification. Systematic Entomology 37: 635–656. 10.1111/j.1365-3113.2012.00641.x

[B25] Smith TL (1965) External morphology of Larva, Pupa, and Adult of the Wax Moth, *Galleria mellonella* L. Journal of the Kansas Entomological Society 38: 287–310.

[B26] Song J-H Ahn K-J (2018) Species trees, temporal divergence and historical biogeography of coastal rove beetles (Coleoptera: Staphylinidae) reveal their early Miocene origin and show that most divergence events occurred in the early Pliocene along the Pacific coasts. Cladistics 34: 313–332. 10.1111/cla.12206 34649372

[B27] Srivathsan A Meier R (2012) On the inappropriate use of Kimura‐2‐parameter (K2P) divergences in the DNA‐barcoding literature. Cladistics 28: 190–194. 10.1111/j.1096-0031.2011.00370.x 34861755

[B28] Walker F (1866) Supplement 5. List of the Specimens of Lepidopterous Insects in the Collection of the British Museum, London 35: 1535–2040.

[B29] Yang J Yang Y Wu WM Zhao J Jiang L (2014) Evidence of Polyethylene Biodegradation by Bacterial Strains from the Guts of Plastic-Eating Waxworms. Environmental Science & Technology 48: 13776–13784. 10.1021/es504038a 25384056

